# Stromal-vascular fraction content and adipose stem cell behavior are altered in morbid obese and post bariatric surgery ex-obese women

**DOI:** 10.1186/s13287-015-0029-x

**Published:** 2015-04-14

**Authors:** Karina R Silva, Sally Liechocki, João R Carneiro, Cesar Claudio-da-Silva, Clarissa M Maya-Monteiro, Radovan Borojevic, Leandra S Baptista

**Affiliations:** Programa de Pós-graduação em Clínica Médica, Universidade Federal do Rio de Janeiro, Rio de Janeiro, RJ 21941-913 Brazil; Núcleo Multidisciplinar de Pesquisa UFRJ – Xerém em Biologia (Numpex-Bio), Universidade Federal do Rio de Janeiro, Polo Xerém, Duque de Caxias, RJ 25245-390 Brazil; Programa de Bioengenharia, Diretoria de Programas, Instituto Nacional de Metrologia, Qualidade e Tecnologia (Inmetro), Duque de Caxias, RJ 25250-020 Brazil; Laboratório de Imunofarmacologia, Instituto Oswaldo Cruz (Fiocruz), Rio de Janeiro, RJ 21.040-900 Brazil; Departamento de Nutrologia do Hospital Universitário Clementino Fraga Filho, Universidade Fereal do Rio de Janeiro, Rio de Janeiro, RJ 21941-913 Brazil; Serviço de Cirurgia Plástica, Hospital Universitário Clementino Fraga Filho, Universidade Federal do Rio de Janeiro, Rio de Janeiro, RJ 21941-913 Brazil

## Abstract

**Introduction:**

Subcutaneous adipose tissue is an interesting source of autologous stem cells with a fundamental role in the pathophysiology of obesity, metabolic syndromes and insulin resistance. We hypothesize that obesity could alter the stromal-vascular fraction (SVF) and adipose stem cell (ASCs) functions, which could compromise its regenerative behavior. Furthermore, we aimed to evaluate whether ASCs derived from post bariatric surgery ex-obese women maintain their functions in a similar fashion as do those from individuals who have never been obese.

**Methods:**

The SVF of subcutaneous adipose tissue from control (n = 6, body mass index – BMI - 27.5 ± 0.5 kg/m^2^), obese (n = 12, BMI 46.2 ± 5.1 kg/m^2^) and post bariatric surgery ex-obese (n = 7, initial BMI 47.8 ± 1.3 kg/m^2^; final BMI 28.1 ± 1.1 kg/m^2^) women were isolated and evaluated by flow cytometry. ASCs were tested for lipid accumulation by perilipin, adipose differentiation-related protein (ADRP) and Oil Red O staining after adipogenic stimulus. The cytokines secreted by the ASCs and after lipid accumulation induction were also evaluated.

**Results:**

The subcutaneous adipose tissue of obese and post bariatric surgery ex-obese women was enriched in pericytes (p = 0.0345). The number of supra-adventitial cells was not altered in the obese patients, but it was highly enriched in the post bariatric surgery ex-obese women (p = 0.0099). The ASCs of the post bariatric surgery ex-obese patients secreted more MCP-1 (monocyte chemoattractant protein-1; p = 0.0078). After lipid accumulation induction, the ASCs of the patients in all groups secreted less IL-6 than the ASCs with no adipogenic stimulus (p < 0.0001). Obese ASCs with lipid accumulation secreted the highest amount of IL-6 (p < 0.001) whereas the ASCs from the controls secreted the highest amount of adiponectin (p < 0.0001). The ASCs from the post bariatric surgery ex-obese patients showed the highest levels of lipid accumulation whereas those from the obese women had the lowest levels (p < 0.0001).

**Conclusions:**

SVF content and ASC behavior are altered in the subcutaneous adipose tissue of morbid obese women; these changes are not completely restored after bariatric surgery-induced weight loss. The cellular alterations described in this study could affect the regenerative effects of adipose stem cells. Further investigations are required to avoid jeopardizing the development of autologous stem cell-based therapies.

## Introduction

Subcutaneous adipose tissue is an interesting source of autologous stem cells for cell-based therapies because of its accessibility, quantity and ease of harvest during aesthetic lipoaspiration procedures [1]. In addition, multiple studies have shown the beneficial effects of subcutaneous fat stem cells in tissue repair, regeneration and immunomodulation via paracrine mechanisms [2-4]. Subcutaneous adipose tissue also has a fundamental role in the pathophysiology of obesity, metabolic syndromes and insulin resistance because a secretory source of adipokines is involved in the inflammatory scenario, such as leptin, adiponectin, interleukin (IL)-6 and IL-8 [5]. Adipocytes and cells from the stromal vascular fraction (SVF) contribute to the secretory function of adipose tissue [6-8]. Although adipocytes are the main source of hormones such as leptin and adiponectin, inflammatory cytokines are mostly secreted by stromal vascular cells [9,10].

The SVF of fat is composed of pericytes, supra-adventitial cells, endothelial cells, fibroblasts and macrophages [11]. Within the adipose tissue, cells with regenerative potential are identified as pericytes (CD45^−^CD146^+^CD34^−^ cells), which reside in small vessels, and supra-adventitial cells (CD45^−^CD146^−^CD34^+^ cells), which dwell in larger vessels with preadipocyte characteristics [12]. SVF cells can be isolated by the enzymatic digestion of adipose tissue and centrifugal separation. Once placed into tissue culture, SVF cells are further separated based on adherence to plastic and culture expansion. Most of the remaining cells are pericytes and supra-adventitial cells, which are now referred to as adipose stem cells (ASCs) [13].

It is well documented that obesity induces an accumulation of macrophages in the adipose SVF. These recruited macrophages contribute to chronic inflammation because of the production of proinflammatory molecules, which is typical of M1 or classically activated macrophages [9,14]. Infiltrated macrophages differ from adipose tissue resident macrophages, called M2 macrophages, which are in an alternatively activated state with anti-inflammatory characteristics [15,16]. Because complete SVF transplant is considered an approach for therapeutic purposes [17-19], it is important to evaluate whether obesity modifies the composition of the progenitor compartment of adipose SVF. Bariatric surgery is commonly used for morbid obesity treatment and leads to massive weight loss. After weight loss stabilization, postbariatric surgery ex-obese patients present residual subcutaneous adipose tissue whose physiology is not yet fully understood. Based on our previous results showing a considerable alteration on the subcutaneous adipose tissue vascular tree [20], we hypothesized that massive weight loss is not enough to recover the nonobese composition of the adipose SVF.

ASCs were found to have an anti-inflammatory effect *in vitro* [21,22], and the paracrine effects of ASCs and adipose SVF cells as regulators of immune tolerance *in vivo* are being widely investigated, opening new therapeutic promise to treat autoimmune diseases [4,18,22-25]. The disruption of adipose tissue homeostasis and stress conditions, as found in diabetes, alters ASC function [26-28], and the treatment of multiple sclerosis with autologous murine ASCs showed no improvement on the disease progression [29]. Therefore, we also hypothesize that the inflammatory scenario established during obesity could alter ASC function in terms of inflammatory cytokine secretion, which could compromise its regenerative behavior. Furthermore, it is important to know whether ASCs derived from postbariatric surgery ex-obese women maintain their functions similarly to those from individuals who have never been obese.

To achieve this goal, the SVF of human abdominal subcutaneous adipose tissue from control, obese and postbariatric surgery ex-obese patients were isolated to evaluate the distribution of the SVF cell populations of subcutaneous adipose tissue and to obtain and characterize ASCs, focusing on functional assays – adipokine secretion and the capacity to accumulate lipids. This study will help to guide the proper use of stem cells derived from obese and postbariatric surgery ex-obese patients for autologous cellular therapy approaches in regenerative medicine. In addition, this study could increase our understanding of how ASCs participate in the development of obesity and their behavior after weight loss induced by bariatric surgery.

This study addresses the following hypotheses: massive weight loss is not sufficient to recover the nonobese composition of the adipose SVF; the inflammatory scenario established during obesity could alter ASC functions in terms of inflammatory cytokine secretions, which could compromise its regenerative behavior; and ASCs derived from postbariatric surgery ex-obese patients could be different to those from individuals who have never been obese.

## Methods

### Patients and tissue harvesting

Abdominal subcutaneous adipose tissue fragments were obtained from nondiabetic female obese patients, postbariatric surgery ex-obese patients and control patients. The obese group included patients with morbid obesity, with a body mass index (BMI) >40 kg/m^2^ (*n* = 12, age = 37.3 ± 13.4 years, BMI = 46.2 ± 5.1 kg/m^2^). Three of the patients presented systemic arterial hypertension. A small fragment of the subcutaneous adipose tissue from these patients was excised during bariatric surgery. The bariatric surgery-induced weight loss patients or the postbariatric surgery ex-obese patients who were included in this study had lost weight during the 2 to 4 years following bariatric surgery, reaching a BMI between 25.0 and 29.9 kg/m^2^ (*n* = 7, age = 45 ± 8.3 years, initial BMI = 47.8 ± 1.3 kg/m^2^; final BMI = 28.1 ± 1.1 kg/m^2^). Because residual subcutaneous adipose tissue remained after the surgery, resulting in flaccid skin due to major weight loss, these patients underwent abdominal plastic surgery, in which a fragment of the subcutaneous adipose tissue was excised. Although the studied tissues of the obese and postbariatric surgery ex-obese patients were not paired samples (presurgery and postsurgery samples), they all followed the same Morbid Obesity Program of the Clementino Fraga Filho University Hospital. All of the obese and postbariatric surgery ex-obese patients were nondiabetic females and had similar BMI values when they had morbid obesity. Nondiabetic patients without previous history of morbid obesity and with a BMI between 25.0 and 29.9 kg/m^2^ (*n* = 6, age = 47.2 ± 11.0 years, BMI = 27.5 ± 0.5 kg/m^2^) were included as a control group and had a fragment of their subcutaneous adipose tissue excised during abdominal plastic surgery performed for aesthetic purposes. This study was approved by the Research Ethics Committee of the Clementino Fraga Filho University Hospital, Federal University of Rio de Janeiro*,* Brazil (Protocols 145/09 and 076/10), and informed consent was obtained from all individual participants included in the study.

### Isolation of stromal vascular fraction and adipose stem cell culture

Subcutaneous adipose tissue fragments from obese patients, postbariatric surgery ex-obese patients and control patients were cut into small pieces and incubated with 1 mg/ml collagenase type II (Sigma Chemical, St. Louis, MO, USA) in a shaking water bath at 37°C for 40 minutes. The mature adipocytes were eliminated by centrifugation (400 × *g*, room temperature, 15 minutes), and the pellet was resuspended for clearing the tissue residues on 100 μm strainers. After counting, the cells were separated for flow cytometry analyses of stromal vascular cells or plated to expand *in vitro* ASCs in tissue culture flasks with low-glucose Dulbecco's modified Eagle's medium (DMEM; LGC, Cotia, Brazil) supplemented with 10% fetal bovine serum (FBS; Cultlab, Campinas, Brazil), 100 U/ml penicillin and 100 μg/ml streptomycin. The cultures were maintained at 37°C in a humid atmosphere with 5% carbon dioxide, and the medium was changed every 3 to 5 days until the cell monolayer reached confluence. At confluence, the cells were harvested with 0.125% trypsin (Gibco, Rockville, MD, USA) and 0.78 mM ethylenediamine tetraacetic acid (Gibco). After harvesting with trypsin, the cells were analyzed by flow cytometry or replated for lipid accumulation and secretory analyses.

### Flow cytometry assay

The cells isolated from the adipose tissue of the obese (*n* = 12), postbariatric surgery ex-obese (*n* = 7) and control (*n* = 6) patients were monitored by flow cytometry for surface marker expression at the moment they were isolated (SVF) and after culture (ASCs). The cell suspensions were washed with phosphate-buffered saline containing 3% bovine serum albumin and incubated for 30 minutes at 4°C with monoclonal antibodies conjugated with fluorescent dyes: CD45–fluorescein isothiocyanate, CD31–phycoerythrin (PE), CD146–PE, CD14–PE, CD34–PE-cyanin 5, CD206–PE-cyanin 5, CD73–peridin chlorophyll and/or CD90–PE (all from BD Biosciences, Franklin Lakes, NJ, USA). Subsequently, the cells were washed with phosphate-buffered saline containing 3% bovine serum albumin. Incubation with FACS lysing solution (BD Biosciences) at room temperature in the absence of light for 10 minutes and with 7-actinomycin D (BD Biosciences) was performed on the SVF cell suspensions. The cells that were stained with a single antibody coupled with a fluorescent dye (fluorescein isothiocyanate, PE, or PE-cyanin 5) were acquired for compensation purposes. For each tube, 100,000 and 20,000 events of SVF and ASC samples, respectively, were acquired in a FACS Calibur (BD Biosciences). The flow cytometry analyses were performed using the software FACS Diva 8.0 (BD Biosciences, Franklin Lakes, NJ, USA). On fresh samples from the morbid obese, postbariatric surgery ex-obese and control patients, a gate was set to include only viable cells (7-actinomycin D negative cells). Pericytes, supra-adventitial and endothelial progenitor cells were identified according to Zimmerlin and colleagues [12]. Resident adipose tissue macrophages were identified among the hematopoietic cells (CD45) by the expression of CD14 and CD206 (mannose receptor) [16]. Cell quantification was expressed as the absolute number of cells per gram of processed adipose tissue.

In the culture-expanded cells (ASCs), the expression of CD34, CD73 and CD90 was given as a percentage of positive cells among the 20,000 events acquired for each sample (*n* = 3 for each group).

### Lipid accumulation stimulus

Induction lipid accumulation was performed by seeding 2 × 10^4^ ASCs in each well of a 48-well plate (in triplicate), cultured with inductive media for up to 6 weeks; the medium consisted of DMEM with 10% FBS, 10 μM insulin, 0.5 mM isobutylmethylxanthine, 1 μM dexamethasone, and 200 μM indomethacin (Sigma). After 3 weeks of induction, the cells were maintained in 0.220 ml/well DMEM with 2% FBS and incubated for 24 hours. After this period, the supernatant was collected and frozen at –80°C, and the monolayer was fixed in 10% buffered formaldehyde for 60 minutes. Lipid accumulation was assessed using Oil Red O staining (Sigma), adipose differentiation-related protein (ADRP) and perilipin detection.

#### Oil red O staining

After the induction to lipid accumulation, the fixed cells were stained with 5 mg/ml Oil Red O in 70% ethanol for 1 hour. Overstaining was washed with buffered phosphate saline. The stain was dissolved for 10 minutes with isopropanol, and the absorbance of the eluted stain was measured in a spectrophotometer at 490 nm. The absorbance of the induced cultures was subtracted from the values obtained with the noninduced cultures. Three replicates for each patient condition (obese, postbariatric surgery ex-obese and control) in five independent experiments were analyzed.

#### Immunofluorescence for adipose differentiation-related protein and perilipin

After the induction to lipid accumulation, the fixed cells were permeabilized with 0.2% Triton X-100 (Merck Millipore, Darmstadt, Germany), followed by incubation with 0.2% monkey serum for 20 minutes to prevent the nonspecific binding of immunoglobulins. An overnight incubation at 4°C was performed with anti-ADRP (1:1,200; Fitzgerald, Acton, MA, USA) and anti-perilipin (1:500; Biorbyt, Cambridge, UK). For the anti-ADRP and anti-perilipin primary antibodies, the secondary antibody staining was performed using Alexa 488-conjugated anti-monkey IgG (Fitzgerald) and cyanin 3-conjugated anti-rabbit (Fitzgerald), respectively, for 1 hour at room temperature. The slides were mounted in a commercial Fluoromount Mounting Medium that contained 4′,6-diamidino-2-phenylindole for nuclear detection (Sigma) and were examined using fluorescence microscopy followed by deconvolution (Olympus, Shinjuku, Tóquio, Japão). The samples incubated without the primary antibodies but with the secondary antibodies were used as the negative controls.

### Cytokine secretion of adipose stem cells

To evaluate cytokine secretion by unstimulated ASCs, the cells were seeded in a 48-well plate (in triplicate, 2 × 10^4^ ASCs in each well) and incubated for 24 hours in DMEM with 2% FBS. To evaluate how the cells responded to a proinflammatory stimulus, 0.5 μg/ml lipopolysaccharide (LPS) was added to the incubating medium. After 3 weeks of adipogenic induction, the adipogenic induced ASCs (in triplicate) were also incubated for 24 hours in DMEM with 2% FBS. After the incubation period, the supernatant of each replicate was collected individually and frozen at −80°C.

The release of cytokines, chemokines and growth factors in the conditioned medium of the unstimulated, LPS-stimulated and adipogenic induced cells was measured using two multiplex immunoassay detection systems capable of analyzing levels of Bio-PlexPro™ human cytokine 17-plex and levels of Human Adipocyte Panel (Merck Millipore) according to the manufacturer’s recommendations. Three replicates for each patient condition (obese, postbariatric surgery ex-obese and control) in three independent experiments were analyzed.

### Statistical analysis

A nonparametric one-way analysis of variance test followed by Dunn’s test was used for comparisons among the control, obese and postbariatric surgery ex-obese patients in the flow cytometry, adipogenic and cytokine secretion assays. Student’s *t* test was used to compare LPS treatment or adipogenic induced cells with unstimulated cells. The results in the graphs are expressed as the mean ± standard error of the mean, and *P* <0.05 was considered statistically significant. The analyses were performed using the software GraphPad Prism 5.0 (GraphPad Software, San Diego, CA, USA).

## Results

### The stromal vascular fraction of obese and postbariatric surgery ex-obese patients was enriched with pericytes and supra-adventitial cells

**Figure 1:**
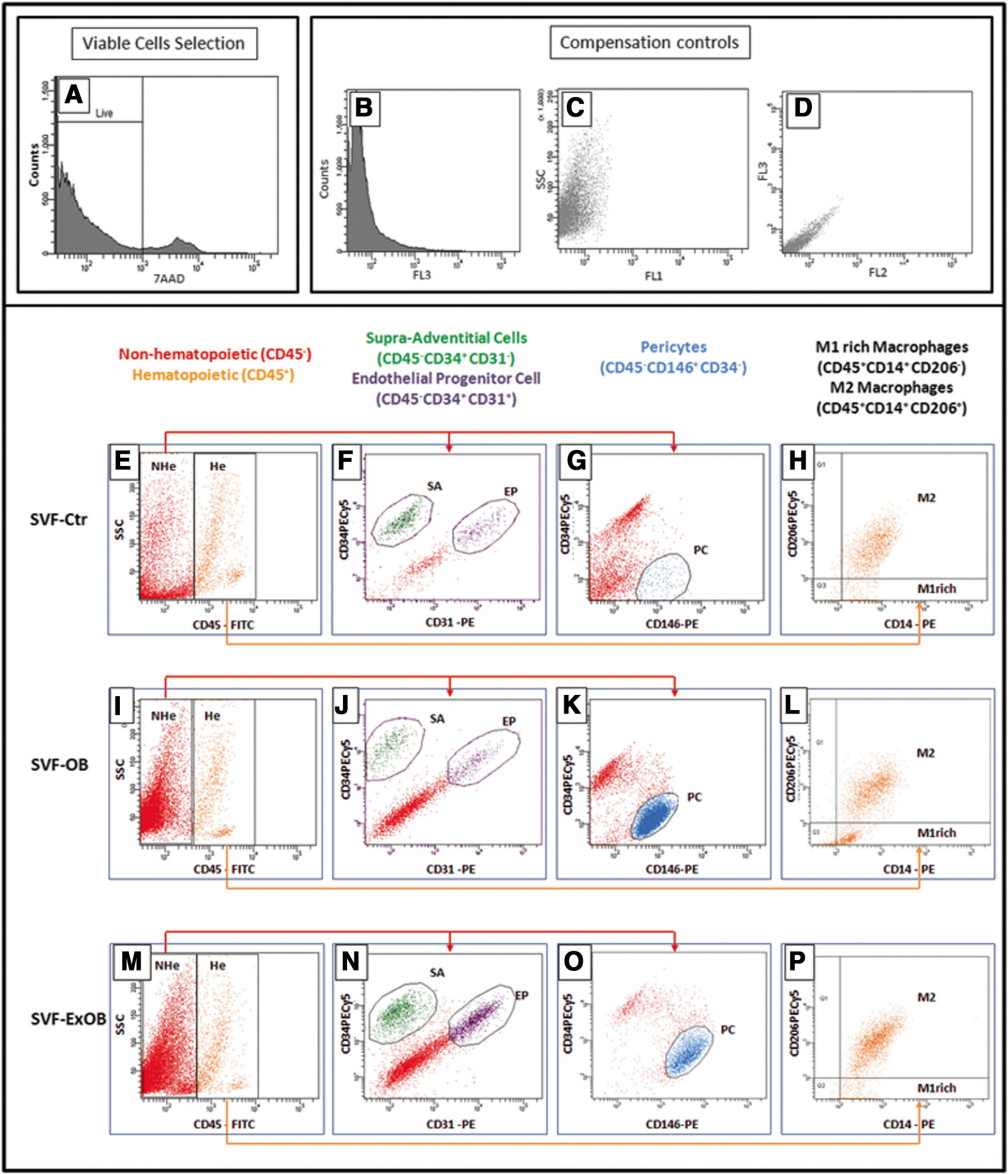
Classification of perivascular and hematopoietic populations for analytical flow cytometry. Stromal vascular fraction (SVF) of adipose tissue from the control, obese and postbariatric surgery ex-obese patients were analyzed by flow cytometry. The viable cells identified by 7-actinomycin D (7AAD) exclusion **(A)** were first analyzed according to CD45 expression **(E, I, M)**. The gates were set on nonhematopoietic (CD45^neg^; NHe) and hematopoietic (CD45^pos^; He) cells. The CD45^neg^ cells (red) were further analyzed for CD34 and CD31 expression **(F, J, N)** or CD34 and CD146 expression **(G, K, O)**. A green gate was set to identify the subset of supra-adventitial cells (SA), which were CD34^pos^CD31^neg^, whereas the endothelial progenitor cells (EP) were identified as CD34^pos^CD31^pos^ (purple gate). A blue gate was set on the CD146^pos^CD34^neg^ cells to identify the pericytes. The mono-macrophage cells were analyzed among the He cells **(H, L, P)**. Adipose tissue resident macrophages were identified as CD14^pos^CD206^pos^ cells (upper right quadrant). A population of CD14^pos^CD206^neg^ was also identified (lower right quadrant). **(B, C, D)** Compensation controls of fluorescence detection. Ctr, control; EP, endothelial progenitor cell; ExOB, postbariatric surgery ex-obese; FITC, fluorescein isothiocyanate; M1rich, enriched in M1 (classically activated) macrophages; M2, alternatively activated macrophages; OB, obese; PC, pericytes; PE, phycoerythrin; PECy5, PE-cyanin 5; SA, supra-adventitial cells.

To evaluate how obesity and weight loss may affect the distribution of SVF subpopulations in subcutaneous adipose tissue, independent samples from obese, postbariatric surgery ex-obese and control patients were analyzed by flow cytometry. A representative sample of each condition is shown in Figure 1. 7-Actinomycin D staining distinguishes nonviable cells present in adipose tissue digests (Figure 1A). The viable cells (negative for 7-actinomycin D) were analyzed for the presence of the classifying markers (CD45, CD146, CD34, CD31, CD14 and CD206) described previously [12,16]. Hematopoietic and nonhematopoietic cells were identified according to the expression of the panhematopoietic marker CD45 (Figure 1E,I,M). The nonhematopoietic cells (CD45^neg^) contained supra-adventitial cells (CD34^pos^CD31^neg^; Figure 1E,J,N), endothelial progenitor cells (CD34^pos^CD31^neg^; Figure 1E,J,N) and pericytes (CD146^pos^CD34^neg^; Figure 1G,K,M).

Subsequently, we analyzed the mono-macrophage CD14^pos^ populations among the hematopoietic cells, according to the expression of CD206 (Figure 1H,L,P). Two mono-macrophage subpopulations were identified: CD14^pos^CD206^pos^ cells, called alternatively activated (M2) macrophages; and CD14^pos^CD206^neg^ cells, which include classically activated macrophages (M1).

**Figure 2:**
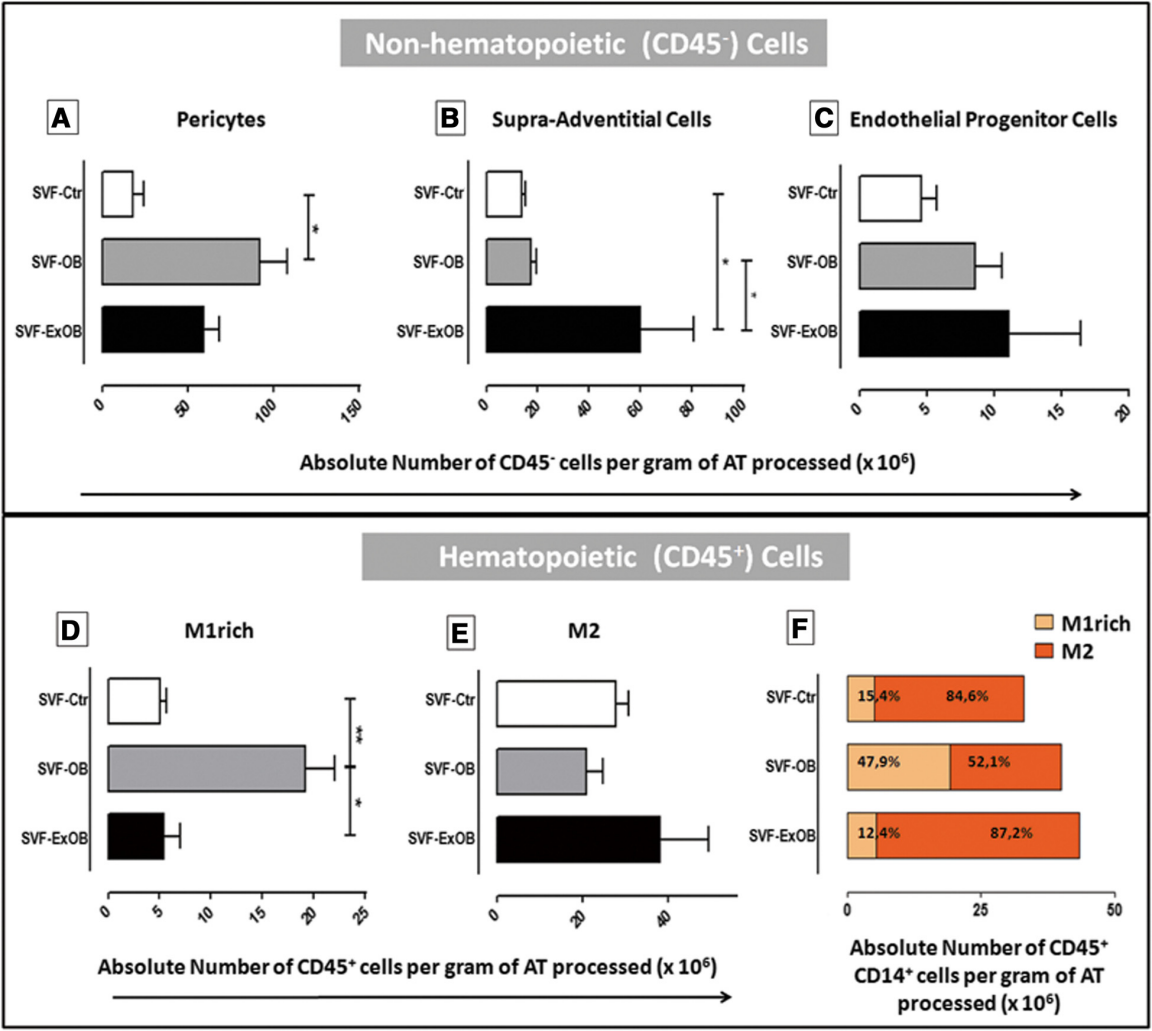
Different stromal vascular fraction contents of adipose tissue from obese, postbariatric surgery ex-obese and control patients. Quantitative data for the flow cytometry analysis show the different distributions of the nonhematopoietic **(A, B, C)** and hematopoietic **(D, E)** cell subsets. The number of cells per adipose tissue (AT) gram from each subpopulation analyzed is expressed as the mean ± standard error of the mean. **(F)** The ratio of CD206^−^ and CD206^+^ cells is expressed. Analysis of variance tests comparing the cell numbers were performed. *P* values from the statistical tests: *P <0.05. Ctr, control; ExOB, postbariatric surgery ex-obese; M1rich, enriched in M1 (classically activated) macrophages; M2, alternatively activated macrophages; OB, obese; SVF, stromal vascular fraction.

Pericytes were most prevalent in the obese patients; additionally, the postbariatric surgery ex-obese patients presented a higher number of pericytes per gram of adipose tissue than the controls (Figure 2A). Interestingly, the adipose tissue from the postbariatric surgery ex-obese patients was the most enriched with supra-adventitial cells (Figure 2B). No significant changes were identified for endothelial progenitor cells (Figure 2C).

The CD45^pos^CD14^pos^CD206^neg^ population, which contains M1 macrophages, was clearly enriched in the obese patients and returned to normal levels after massive weight loss (Figure 2D). No statistically significant difference was observed in the M2 macrophage content (Figure 2E). The proportion of the two macrophage subpopulations (M1 and M2) was similar in the obese adipose tissue, whereas M2 macrophages were predominant in the control and postbariatric surgery ex-obese patients (Figure 2F). However, the postbariatric surgery ex-obese adipose tissue showed a slight increase in the absolute number concomitant to a predominance of M2 (Figure 2E,F).

### Obesity alters ASC cytokine secretion profiles, which are not completely restored after massive weight loss

**Figure 3:**
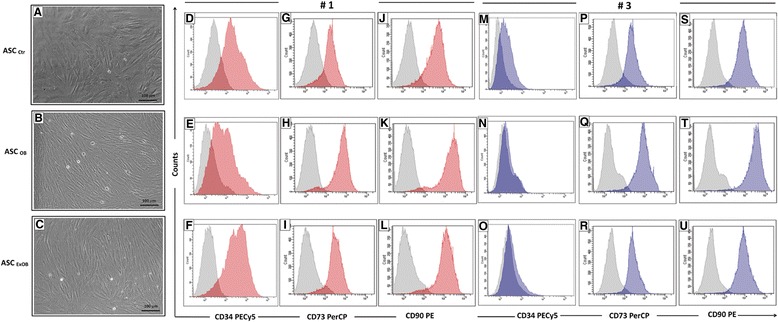
Phenotypical characterization of adipose stem cells from the control, obese and postbariatric surgery ex-obese patients. Adipose stromal/stem cells (ASCs) from the subcutaneous adipose tissue of patients for all conditions showed fibroblastoid cells (**A**, **B**, **C**, phase contrast, 100× magnification). Histograms show the ASC surface expression of CD34 **(D, E, F, M, N, O)**, CD73 **(G, H, I, P, Q, R)** and CD90 **(J, K, L, S, T, U)** of the cells cultivated at passage 1 (**D** to **L**) and expanded up to passage 3 (**M** to **U**). Histograms of the ASCs from the control **(D, G, J, M, P, S)**, obese **(E, H, K, N, Q, T)** and postbariatric surgery ex-obese **(F, I, L, O, R, U)** samples are representative of three independent experiments. Gray histogram, negative control; red histogram, expression at passage 1; blue histogram, expression at passage 3. Quantitative results are summarized in Table 1. *P* values from the analysis of variance tests comparing percentage numbers: *P = 0.038. Ctr, control; ExOB, postbariatric surgery ex-obese; OB, obese; PE, phycoerythrin; PECy5, PE-cyanin 5; PERCP, peridin chlorophyll.

After *in vitro* cultivation, the ASCs from the subcutaneous fragments of the obese, postbariatric surgery ex-obese and control patients had similar fibroblastoid morphology (Figure 3A,B,C). A flow cytometry analysis of ASCs at passage 1 for the supra-adventitial/preadipocyte marker CD34 (Figure 3D,E,F) and for the *in vitro* mesenchymal markers CD73 (Figure 3G,H,I) and CD90 (Figure 3J,K,L) revealed differences only concerning CD34 expression. The quantitative data from the flow cytometry analyses (Table 1) showed that the ASCs from the obese patients had the lowest number of CD34^pos^cells whereas this value was similar in postbariatric surgery ex-obese and control patients. As expected, the ASCs from all conditions at passage 3 showed a more homogeneous population (Figure 3M to U). All of the following experiments were performed using the cells at passage 3.

**Table 1:** **Quantification of CD34, CD73 and CD90 expression by adipose stem cells**

	**CD45** ^**–**^ **CD34** ^**+**^ **(%)**				**CD45** ^**–**^ **CD73** ^**+**^ **(%)**				**CD45** ^**–**^ **CD90** ^**+**^ **(%)**			
	**Passage 1** ^**a**^		**Passage 3**		**Passage 1**		**Passage 3**		**Passage 1**		**Passage 3**	
	**Mean**	**SD**	**Mean**	**SD**	**Mean**	**SD**	**Mean**	**SD**	**Mean**	**SD**	**Mean**	**SD**
ASC Ctr	82	±9.9	1.4	±2.5	83.4	±11.9	93.9	±2.6	88	±10.2	96.5	±1.6
ASC OB	62.01	±15.5	0	±0	87.3	±2.1	93.1	±1.3	92.3	±2.76	97.8	±0.3
ASC ExOB	85.2	±3.1	1.1	±1.9	95	±2.8	94.1	±1.5	96.2	±1.2	96.8	±1.7

ASC, adipose stem cell; Ctr, control; ExOB, postbariatric surgery ex-obese; OB, obese; SD, standard deviation. ^a^Analysis of variance: *P* = 0.038.

**Figure 4:**
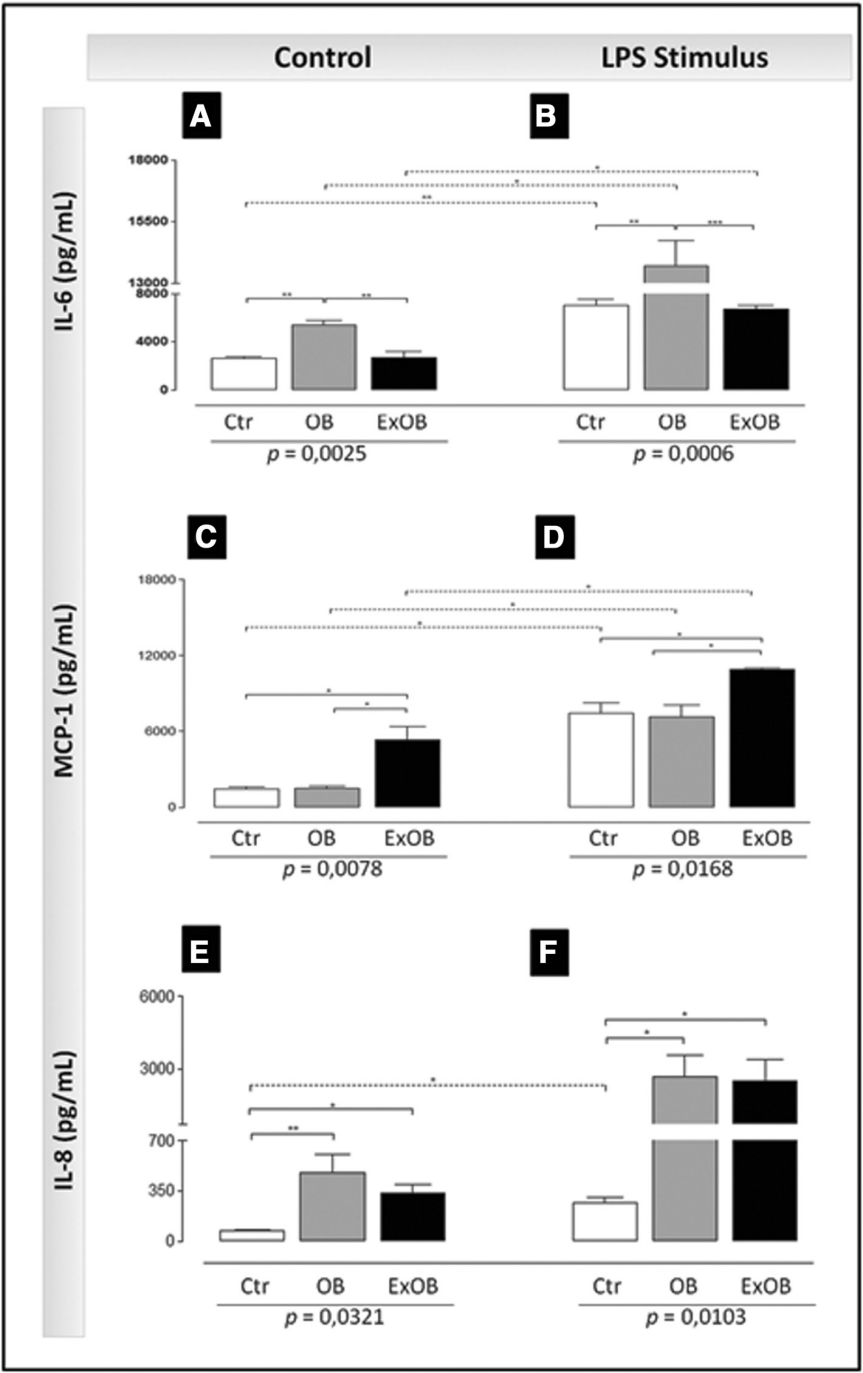
Differential cytokine secretion of adipose stem cells isolated from patients with different conditions. Cytokine secretion in the supernatant of cells cultivated under control conditions **(A, C, F)** and under lipopolysaccharide (LPS) stimuli **(B, D, F)** was evaluated using a multiplex assay. The graphs show data from one representative experiment of three independent experiments. *P* values obtained after the analysis of variance (ANOVA) tests are indicated under each graph. Solid and dotted lines indicate the statistical results of the ANOVA post test and the *t* test, respectively. *P* values from the statistical tests: *P <0.05, **P <0.001. Ctr, control; ExOB, postbariatric surgery ex-obese; IL, interleukin; MCP-1, macrophage chemoattractive protein-1; OB, obese.

We also hypothesized that obesity and massive weight loss could modify ASCs in terms of cytokine secretion, which could represent different paracrine properties. Highly confluent cultures of ASCs were maintained for 24 hours under standard cell culture conditions (control) or LPS stimulation to simulate a proinflammatory stimulus. The quantitative data for cytokine secretion on the supernatant are expressed in Figure 4. Under control conditions, the ASCs from all groups secreted IL-6, IL-8, macrophage chemoattractive protein-1 (MCP-1; Figure 4A,C,E) and Plasminogen activator inhibitor-1 (data not shown). The ASCs from the morbid obese patients secreted more proinflammatory cytokines such as IL-6 (Figure 4A) and IL-8 (Figure 4E) than the control ASCs. The postbariatric surgery ex-obese patients showed a 3.7-fold greater secretion of MCP-1 compared with the controls. Under proinflammatory stimulation with LPS, the ASCs from all conditions responded by enhancing cytokine secretion of IL-6, IL-8 and MCP-1 (Figure 4B,D,F).

### Obesity decreases ASC lipid accumulation capacity and adiponectin secreted levels

**Figure 5:**
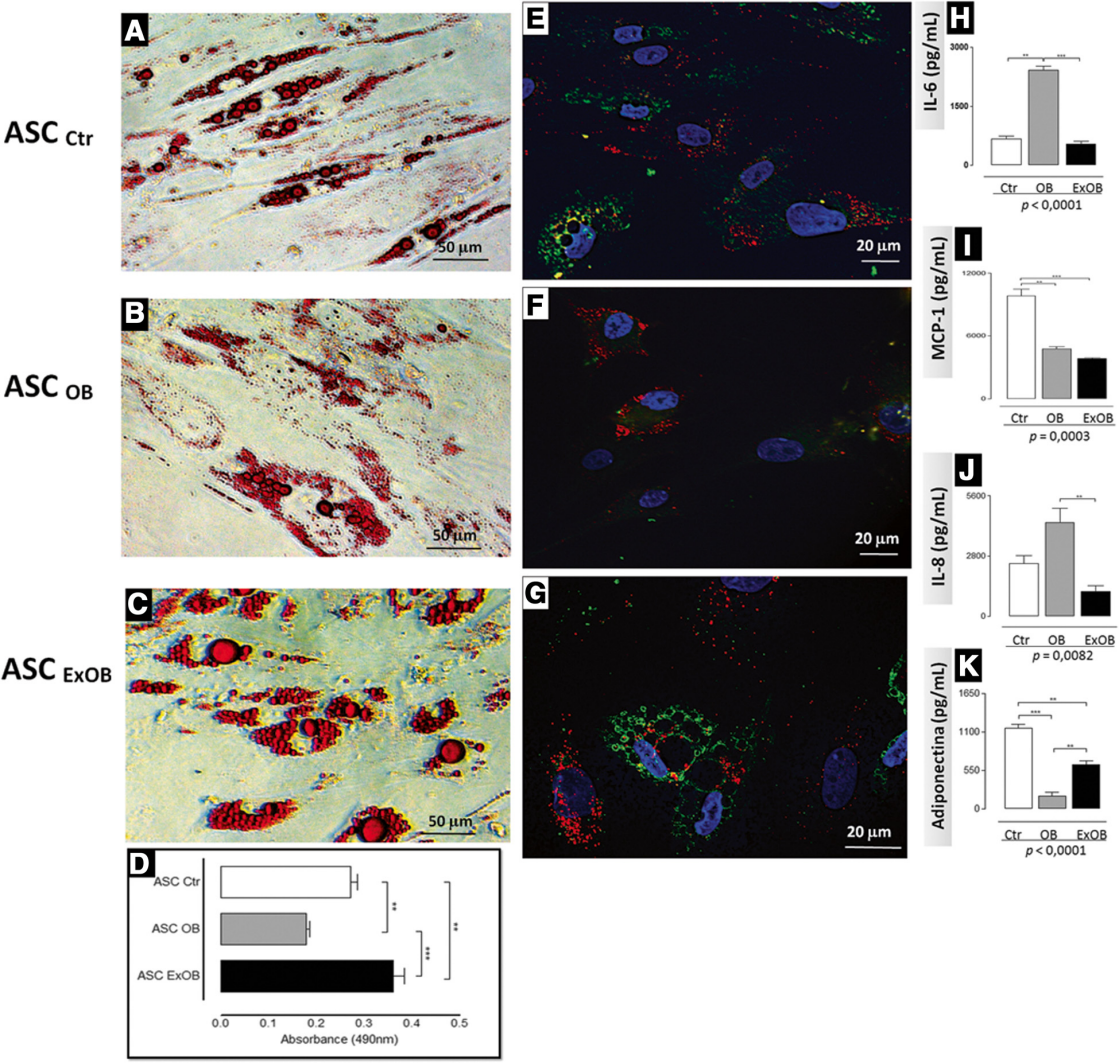
Differential lipid accumulation capacity and cytokine secretion of induced adipose stem cells from obese, postbariatric surgery ex-obese and control patients. The adipose stem cells (ASCs) were cultured under inductive medium to accumulate lipids for up to 3 weeks, after which lipid accumulation was accompanied by Oil Red O staining **(A, B, C)** or by staining with perilipin and adipose differentiation-related protein **(E, F, G)**. The total Oil Red O dye captured by the lipid droplets was measured in a spectrophotometer. **(D)** Representative data from five independent experiments, expressed as the mean ± standard error of the mean, which represents the quantitative results of the Oil Red O staining. Analysis of variance (ANOVA) tests comparing the absorbances were performed (*P <*0.0001). The supernatant of the induced cells was evaluated for cytokine secretion. **(H)** Interleukin (IL)-6, **(I)** macrophage chemoattractive protein-1 (MCP-1), **(J)** IL-8 and **(K)** adiponectin. ANOVA tests comparing the secretions were performed. *P* values from the statistical tests: **P <0.001, ***P <0.0001. **(A, B, C)** Optical microscopy, bar size: 50 μm. **(D, E, F)** Fluorescence laser confocal microscopy, bar size = 20 μm. Ctr, control; ExOB, postbariatric surgery ex-obese; OB, obese.

The ability of ASCs to accumulate lipids *in vitro* was evaluated among the studied groups. The ASCs from obese patients showed the lowest lipid accumulation capacity (Figure 5A,B,C,D). The quantitative data for the total amount of Oil Red O stain captured by lipid droplets are expressed in Figure 5D. Qualitatively, the lipid droplets of cells from the postbariatric surgery ex-obese patients showed a prevalence of perilipin staining (Figure 5G), which is typical of adipocyte maturation [30], whereas those from the obese patients’ ASCs showed a prevalence of ADRP (Figure 5F), which is usually found in earlier phases of adipogenesis [30].

The cytokine secretion profile was also evaluated after 3 weeks of *in vitro* adipogenic stimulus (Figure 5H,I,J,K). The ASCs showing lipid accumulation secreted less IL-6 (Figure 5H) than the corresponding ASCs with no lipid accumulation (Figure 4A), as described recently [31]. In addition, higher amounts of IL-6 (Figure 5H) and IL-8 (Figure 5J) were secreted by lipid accumulation-stimulated obese ASCs than by the control and postbariatric surgery ex-obese ASCs. Interestingly, MCP-1 secretion was the highest in the ASCs with lipid accumulation derived from the control patients (Figure 5I). In patients of all conditions, the secretion of the anti-inflammatory cytokine adiponectin was only detected in cells after an adipogenic stimulus. The ASCs showing lipid accumulation of the control showed the highest adiponectin secretion, whereas the postbariatric surgery ex-obese samples had lower adiponectin secretion, and the obese samples had the lowest secretion levels (Figure 5K).

## Discussion

Herein we show that SVF content and ASC behavior *in vitro* are altered in severely obese women. In addition, massive weight loss induced by treatment with bariatric surgery does not recover the normal cell physiology of ASCs in terms of adipogenesis and cytokine secretion.

A flow cytometry analysis performed in freshly isolated cells revealed that obese and ex-obese subcutaneous fat contains an increased number of pericytes. These results may be related to changes in the blood vessels’ content that occurs during obesity [32-34]. A higher frequency of pericytes was found in the ex-obese patients compared with the control patients; this result is consistent with our previous study, which described that the number of small vessels is up to twofold times higher compared with the control patients [20]. Furthermore, there was also increasing evidence for the key role of stem cells in obesity [35]. In a mouse model, tumor necrosis factor alpha, which is highly produced in obese adipose tissue, contributes to the recruitment of both macrophages [9] and stem cells [36]. CD146, a pericyte surface marker, is involved in monocyte transendothelial migration during inflammation [37]. The higher frequency of pericytes in obese adipose tissue may therefore result from a cytokine-mediated mobilization of these cells, representing a source of cells available for tissue growth, repair and wound healing.

According to the overflow hypothesis described by Bergman and coworkers [38], the visceral fat compartment increases primarily with moderate fat intake. However, as fat feeding increases, both visceral and subcutaneous fat enlargement occurs. We could presume that this subcutaneous fat tissue enlargement could be because of an increase in the number of pericytes, which are able to generate new preadipocytes [32] that are capable of storing lipids and meeting the amount of fat intake.

We found that the ex-obese patients had fourfold more supra-adventitial cells than the obese patients. Because adipocytes are able to dedifferentiate [39], it could be assumed that during massive weight loss the adipocytes dedifferentiated into supra-adventitial cells because these cells have preadipocyte characteristics [12]. This result was also consistent with our previous study of postbariatric surgery ex-obese subcutaneous fat, showing that the area occupied by robust blood vessels with an adventitial layer is up to two times larger than the control area [20].

Adult stem cells from subcutaneous adipose tissue are in fact regenerative cells with wide use in medicine regenerative approaches. Within the SVF, regenerative cells include pericytes and supra-adventitial cells. Because of the ease of subcutaneous harvesting and the possibility of SVF isolation in the operating room, a variety of clinical trials uses SVF [17,18]. The enrichment of pericytes and supra-adventitial cells found in obese and postbariatric surgery ex-obese adipose tissues, respectively, could have an unknown effect on the regenerative effects of the SVF.

We analyzed mono-macrophage CD14^pos^ populations among hematopoietic cells according to the expression of CD206 (Figure 1H,L,P). Two mono-macrophage subpopulations were identified: CD14^pos^CD206^pos^ cells, called alternatively activated (M2) macrophages; and CD14^pos^CD206^neg^ cells, which include classically activated macrophages (M1). Because of the lack of an appropriate marker that identifies exclusively human M1 macrophages, we could not quantify this population, but the CD45^pos^CD14^pos^CD206^neg^ population, which contains M1 macrophages, is clearly enriched in obese patients, as expected [40,41], and returns to normal levels after massive weight loss (Figure 2D). M1 macrophages recruited to the SVF during obesity development are known to secrete proinflammatory cytokines [9,14], in contrast to the anti-inflammatory state found in M2 resident adipose tissue macrophages. It was recently observed that M2 macrophages were increased in animals during weight loss [42]. In addition, an increase in M2 macrophage counts in human adipose tissue 3 months after gastric surgery was reported [41]. Macrophages with M2 characteristics can play an essential role in tissue homeostasis by preventing inflammation and promoting insulin sensitivity. Our results show that this increase may be persistent because the analyses were performed at least 2 years after bariatric surgery.

Some studies have investigated the cytokine secretion of ASCs isolated from human subcutaneous adipose tissue with different adiposity grades [10,31,43] such as obese and nonobese individuals. An upregulation of inflammatory genes in ASCs isolated from obese subcutaneous adipose tissue has been reported [44]. Accordingly, we found that ASCs from the morbid obese patients secreted more proinflammatory cytokines such as IL-6 and IL-8.

MCP-1 was highly secreted by the postbariatric surgery ex-obese ASCs, and we observed no differences between the control and obese patients. It is well established that macrophages are able to secrete MCP-1, resulting in macrophage mobilization. Recently, Cancello and colleagues demonstrated that the gene expression of human subcutaneous adipose tissue is not fully restored after massive and stable weight loss, suggesting a persistence of obesity molecular characteristics [45]. We can thus postulate that *in vivo* counterparts of postbariatric surgery ex-obese ASCs could also contribute to macrophage recruitment.

The secretion of these cytokines was further upregulated after incubation with a proinflammatory stimulus (LPS). Lee and colleagues showed that tumor necrosis factor alpha induction augmented the secretion of IL-6, IL-8 and MCP-1 of ASCs [43]. In addition, it was demonstrated that LPS also induced ASCs to secrete IL-6, IL-8 and tumor necrosis factor alpha [46], indicating that ASCs are responsive to proinflammatory stimuli by secreting proinflammatory cytokines.

Indeed, it was observed that ASC function is altered when the disruption of tissue homeostasis occurs, such as in diabetes [26-28]. The alterations in ASC function observed in the present study may therefore be a result of the homeostasis disruption found in the obesity inflammatory scenario.

In our previous study, ASCs isolated from postbariatric surgery ex-obese patients had an accelerated lipid accumulation under an adipogenic stimulus compared with the controls [20]. However, in this study, the ASCs from obese patients showed the lowest lipid accumulation capacity. Some studies have shown an inverse correlation between BMI and lipid accumulation capacity. In addition, obesity reduces the differentiation capacity of the adipose ASCs [44-48], and it has been recently proposed that the inflammatory state could be responsible for impaired adipocyte differentiation [49]. Recently, Oñate and colleagues have shown an upregulation of inflammatory genes coinciding with a reduction of stemcellness of obese ASCs [44]. The prevalence of lipid droplets with perilipin expression observed in postbariatric surgery ex-obese cells confirms a precommitment of ASCs with the adipogenic lineage.

Proinflammatory cytokines secreted by macrophages in the adipose tissue during obesity development are known to inhibit the adipogenesis of adipocyte precursor cells *in vitro* [50,51]. A recent study showed that obesity modifies ASC plasticity to differentiate towards adipogenic and osteo-chondrophenotypes in mice [52]. Considering that *in vitro* cultivated ASCs are continuously attracting the attention of clinicians because of their therapeutic potential for regenerative medicine, cell therapy and tissue engineering, it is necessary to better understand the mechanisms by which the differentiation capacity of ASCs are altered in obesity.

Otherwise, the higher lipid accumulation observed in the ASCs of postbariatric surgery ex-obese patients could be explained by the high content of supra-adventitial cells in the SVF, which was observed by flow cytometry. An adipogenic commitment on the SVF along with an altered vascular tree on the adipose tissue of postbariatric surgery ex-obese patients [20] may favor the weight regain and possibly a return to the obese state. Further investigations based on cell sorting of supra-adventitial populations will clarify their pathophysiological contributions.

Adiponectin is an anti-inflammatory cytokine, and its production by adipose tissue is inversely correlated to BMI [53]. Although the control and postbariatric surgery ex-obese patients from the present study had similar BMI levels, ASCs from the postbariatric surgery ex-obese patients showing lipid accumulation had secreted lower levels of adiponectin, reinforcing the fact that postbariatric surgery ex-obese tissues do not restore adipose tissues to the normal scenario.

The cohort of patients included in this study may not necessarily represent the general population in terms of adiposity and socioeconomic characteristics, which may limit the application of our study to the general population. Such subgroup selections should be taken into consideration when comparing our results with other studies because they introduce the potential for bias and confounding.

## Conclusions

Herein, we show that obesity is responsible for an altered subcutaneous adipose tissue SVF composition and ASC behavior *in vitro*. Furthermore, massive weight loss induced by treatment with bariatric surgery does not recover the normal cell physiology of ASCs in terms of lipid accumulation and cytokine secretion. These alterations could affect regenerative effects when using these cells as tools for regenerative medicine. Further molecular investigations after the cell sorting of pericytes and supra-adventitial cells are required to progress our knowledge of obese and postbariatric surgery ex-obese subcutaneous adipose tissues to avoid jeopardizing the development of autologous stem cell-based therapies.

## Abbreviations

ADRP: adipose differentiation-related protein
ASC: adipose stem cell
BMI: body mass index
DMEM: Dulbecco’s modified Eagle’s medium
FBS: fetal bovine serum
IL: interleukin
LPS: lipopolysaccharide
MCP-1: macrophage chemoattractive protein-1
PE: phycoerythrin
SVF: stromal vascular fraction
